# Short-term report of early glaucoma surgery with a clear lens extraction and an intraocular lens, OMNI canaloplasty, and a HYDRUS microstent: a case series in younger patients

**DOI:** 10.3389/fopht.2023.1288052

**Published:** 2024-01-05

**Authors:** Daniel Laroche, Abelard Desrosiers, Chester Ng

**Affiliations:** ^1^ Department of Ophthalmology, Icahn School of Medicine, Mount Sinai, New York, NY, United States; ^2^ Advanced Eye Care New York, New York, NY, United States; ^3^ City University of New York (CUNY) School of Medicine, New York, NY, United States

**Keywords:** OMNI canaloplasty, HYDRUS stent, glaucoma, clear lens extraction, MIGS

## Abstract

**Purpose:**

The purpose of this case series is to report the surgical outcomes from the combination of a clear lensectomy, OMNI® canaloplasty, and a HYDRUS® microstent with an adjacent goniotomy.

**Methods:**

This is a retrospective non-comparative single-center case series of four black patients of African descent with glaucoma who were treated with a clear lensectomy, OMNI canaloplasty, and a HYDRUS microstent with an adjacent goniotomy. The surgeries were performed by an experienced cataract and glaucoma surgeon, Daniel Laroche, MD. The parameters investigated in this study were postoperative intraocular pressure (IOP) and the mean number of preoperative and postoperative medications needed.

**Results:**

The mean age of the four patients was 44.5 years. All patients had a mean postoperative reduction in IOP of 17 mmHg to 12.7 mmHg. The mean number of preoperative medications was 2.2, while the mean number of postoperative medications was 0.3. Potential complications such as hyphema, IOP spikes, or corneal edema were not seen in this series. All patients achieved a lower IOP and stable vision with less refractive error. Patients also experienced improved visual fields, clearer vision, and more open angles.

**Conclusion:**

Clear lensectomy and combined microinvasive glaucoma surgery (MIGS) in patients with primary open-angle glaucoma (POAG) and narrow-angle glaucoma (NAG) results in the safe lowering of IOP. The limitations of this study include the small series size and the retrospective potential for bias. Further research with a larger series and a prospective trial with follow-up should be performed.

## Introduction

Glaucoma is a group of progressive optic neuropathies that is the second leading cause of blindness globally, with an estimated prevalence of 76 million people worldwide in 2020 (1). Moreover, in the year 2040, the number of people with glaucoma is projected to increase to 111.8 million due to the expected significant increase in the number of aged persons (1). Primary open-angle glaucoma (POAG) constituted 58.6 million of the total number of cases in 2020, and this number has increased today, in line with the 2040 projections (1). A sustained increase in intraocular pressure (IOP) has been identified as the primary risk factor and contributor to the optic nerve neuropathy in POAG (2, 3). Elevated IOP has been known to cause compression of the vascular supply to the optic nerve head, leading to chronic ischemia and causing the insidious onset of visual field loss and irreversible blindness (2, 3). The current mainstay treatment of POAG is focused on the management of the IOP through medical and surgical means to achieve the target IOP. Reaching and maintaining the target IOP for a given patient ceases the associated symptoms of glaucoma and progression of visual field loss and can prevent total vision loss (4).

Currently, topical medical therapy, consisting of hypotensive medications that the patient would need to take for life to maintain the target IOP, is the initial treatment for glaucoma (5). These medications are typically β-blockers, α-agonists, carbonic anhydrase inhibitors, or prostaglandin analogs that have been proven to be effective at lowering IOP (5). However, it has been shown that patient persistence and adherence to these eye drops is low, and this has a significant impact on the long-term management of glaucoma patients (6, 7). Looking at IOP management using a surgical approach, many studies have already established that cataract surgery alone is successful at lowering the IOP in POAG patients (8, 9). Poley et al. hypothesized that the replacement of a new lens in cataract surgery returns the anterior chamber (AC) to the position it occupied earlier in a patient’s life (10). Moreover, the anterior lens capsule becomes more posterior due to the surgery, which has the downstream effect of decreasing iridolenticular rubbing and pigment liberation, which in turn results in the obstruction of the trabecular meshwork (11). This also increases the lumen size of Schlemm’s canal, which in turn allows greater aqueous humor drainage (10). Although cataract surgery provides this benefit of lowering IOP, other studies convey that this surgical approach may not be enough to prevent postoperative hypertensive spikes and long-term IOP lowering (12–15).

Recently, it has been shown that early microinvasive glaucoma surgery (MIGS) procedures combined with cataract extraction surgery successfully lowers the IOP in patients with mild to moderate glaucoma and reduces the number of medications needed (16). Moreover, it has been shown that the visual acuity of patients who have undergone this combined procedure has improved (17). Clear lens extraction with intraocular lens (IOL) placement and MIGS have also been shown to lower the IOP with fewer medications required. Combination MIGS with clear lens extraction has been reported (18, 19). In addition to clear lens extraction combined with the MIGS procedure, OMNI® canaloplasty and a HYDRUS® microstent have been reported to lower patients’ IOP and medication use (20, 21). Large studies have conveyed that the mean age at which patients undergo cataract surgery is between 70 years and 80 years (22, 23). The mean age at which patients underwent glaucoma surgery in the “Tube vs. Trabeculectomy” study was 71 years (24). In this study, we report on four cases of young black glaucoma patients, with a mean age of 44.5 years, who were treated using a combination of clear extraction and IOL placement, a HYDRUS microstent, and OMNI viscocanalostomy to lower their IOP and reduce their medication burden.

## Surgical technique

**Figure 1:**
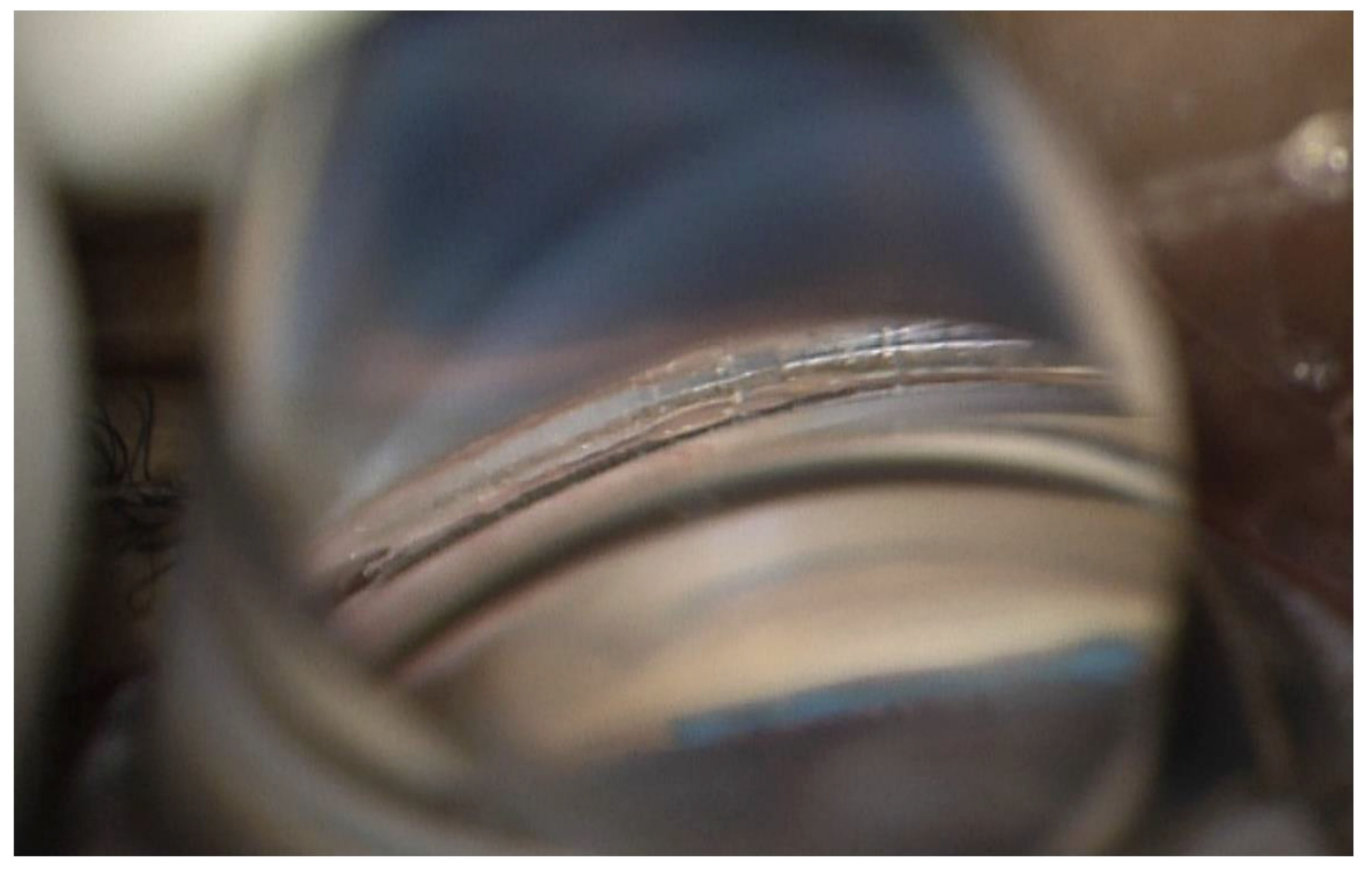
OMNI canaloplasty of Schlemm’s canal after clear lensectomy.

**Figure 2:**
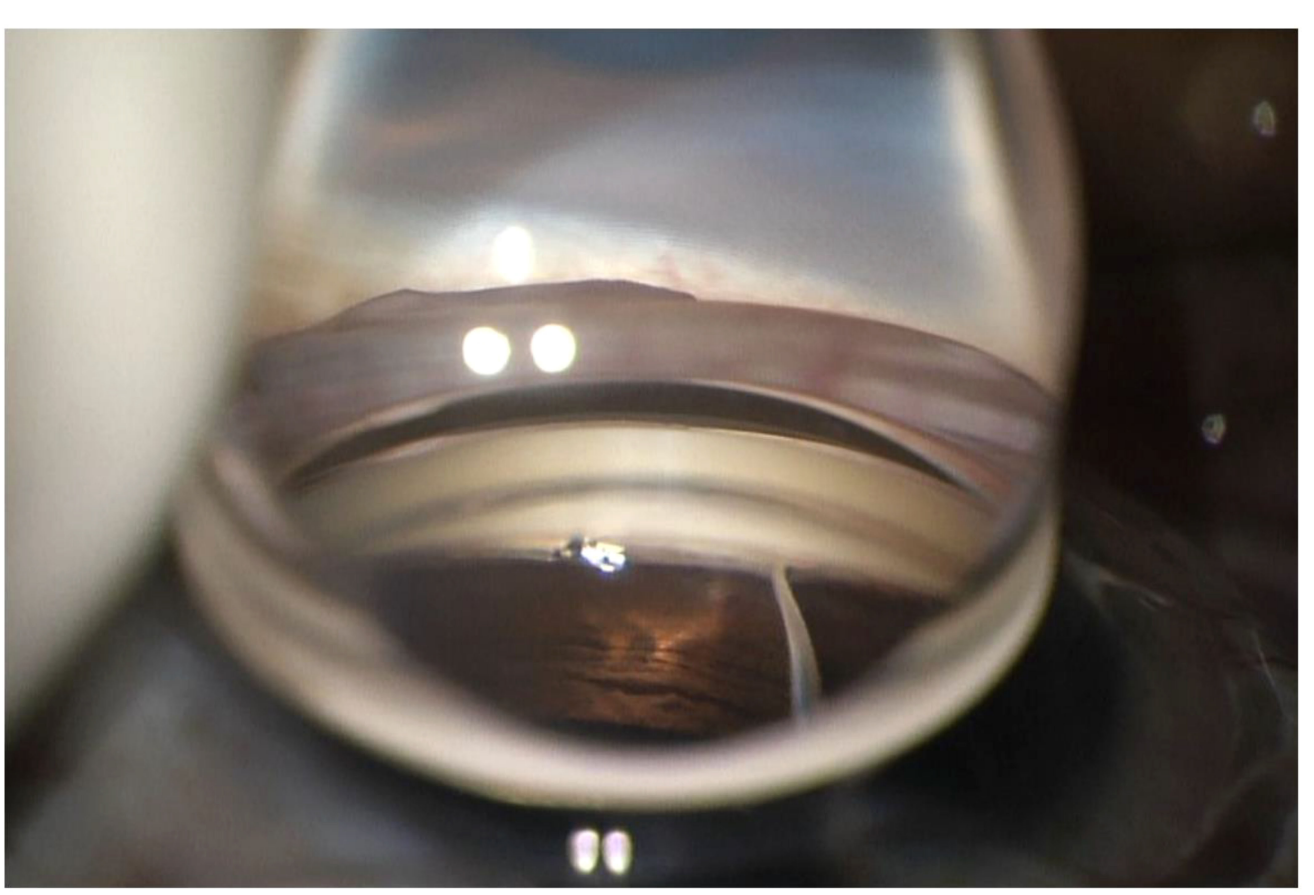
Insertion of a HYDRUS microstent with an adjacent goniotomy with a Sinskey hook.

All patients provided written informed consent. The patients were administered preoperative 1% prednisolone acetate (Allergan, Dublin, Ireland) [four times a day (QID)], 0.5% ketorolac [three times a day (TID)], and 0.3% ofloxacin (Rising, Saddle Brook, NJ, USA) (QID) starting 3 days prior to the surgery. After the eye was prepped with betadine and draped, topical anesthesia was applied, and a clear corneal phacoemulsification was performed with the implantation of an intraocular lens. EndoCoat® (Abbott, Chicago, IL, USA) was placed in the eye to deepen the angle. A paracentesis was made 2.5 clock hours to the right of the corneal incision and EndoCoat was placed on the cornea. The patient’s head was tilted at an angle of approximately 45° away from the surgeon, and the microscope was tilted at an angle of approximately 45° toward the surgeon. A direct goniolens (Katena, Troy Hills, NJ, USA) was placed on the eye, and the microscope was then focused so that a direct view of the nasal angle structures could be obtained. A microvitreoretinal (MVR) blade was used to make an incision through the trabecular meshwork into Schlemm’s canal. The OMNI device (Sight Sciences) was primed and placed into the anterior chamber (AC) through the paracentesis, and the tip of the device was placed inside Schlemm’s canal. The catheter was inserted into Schlemm’s canal and advanced 180 degrees, then slowly withdrawn, leaving behind visoelastic in the canal (**Figure 1**). The HYDRUS® microstent inserter was then passed into the AC through the paracentesis. The tip of the device injector was placed inside Schlemm’s canal (SC) and the microstent was threaded into the canal over a span of approximately 90°. On visual confirmation of the proper device positioning in the canal, the delivery system was withdrawn and the ophthalmic viscosurgical device removed. A Sinskey hook was then used to ensure the microstent was fully advanced into the canal and to perform an adjacent two clock-hour goniotomy (**Figure 2**). The visoelastic was then removed and replaced with a balanced salt solution. An intracameral injection of Vigamox® (Alcon, Geneva, Switzerland), which was diluted with balanced saline solution (50:50; 1 cc), was given during the paracentesis at the end of the procedure. The patient was sat upright at the end of the procedure and was instructed to sleep in a seated or upright position for 4 days to reduce the episcleral venous pressure in the eye and to lessen the risk of blood reflux and hyphema.

## Case study 1

A 42-year-old black female patient with a history of POAG presented for further evaluation. She had no other significant past medical history or past surgical history. She had no known drug allergies. Her medications included latanoprost [right eye (OD) once a day (QD)] for elevated eye pressure. Her visual acuity was 20/20 [both eyes (OU)]. The patient’s refraction value was +0.50 −2.25 × 177 in the OD, and 0.00 −4.25 × 177 in the left eye (OS). Their IOP was 17 in the OD and 16 in the OS. The dark-room gonioscopy revealed that the Shaffer grade II angles were open, but occludable, with the pigmentation being heavier inferiorly than it was superiorly due to the iridolenticular rubbing of the iris against the lens. There was no peripheral anterior synechia detected. The patient’s cup-to-disc ratio (CDR) was 0.65/0.65 in the OD and 0.6/0.6 in the OS. An inferior notch was noted in the OS. Optical coherence tomography of the optic nerve head (OCT–ONH) revealed an average retinal nerve fiber layer (RNFL) thickness of 79 ųm in the OD and 86 ųm in the OS. The Humphrey visual field (HVF) analysis findings revealed a visual field index (VFI) of 91%, with a mean deviation (MD) of −7.14 in the OD and a VFI of 87% with an MD of −8.73 in the OS. The central corneal thickness was 530 ųm in the OD and 540 ųm in the OS. The target IOP for this patient was 10 mmHg–14 mmHg for both eyes.

After discussing options for treatment, the patient decided to undergo glaucoma surgery consisting of clear lens extraction and IOL placement, OMNI viscocanaloplasty, and a HYDRUS stent with an adjacent goniotomy in the OS. The patient agreed to the risks and benefits and underwent the surgery without experiencing any complications. After the surgery, the patient was prescribed prednisolone acetate (QID), ketorolac (TID), and ofloxacin (QID) for the left eye while discontinuing latanoprost, also in the left eye. The patient was instructed to keep their head elevated above waist level for the first week, including when sleeping at night. On the first day post operation, the patient’s IOP was 23 mmHg in the OD and 9 mmHg in the OS. The patient was then instructed to take latanoprost in the right eye. The patient returned 3 weeks post operation, and their IOP was found to be 19 mmHg in the OD and 17 mmHg in the OS. The patient continued latanoprost in the right eye, and their dosing of prednisolone acetate and ketorolac were tapered down. The patient then returned 5 months post operation, at which point their visual acuity was found to be 20/20 in the OD and 20/20 in the OS. Their postoperative refraction value was 0.00 −2.50 × 006 in the OS, resulting in an improvement in refractive error, with a decrease of 1.75 D in astigmatism. The patient’s IOP was stable, at 16 mmHg in the OD and 15 mmHg in the OS. The HVF analysis revealed improved visual fields, with a VFI of 95% and an MD of −5.26 dB in the OD and a VFI of 88% and an MD of −5.96 dB in the OS.

## Case study 2

A 41-year-old black female patient with a past medical history of anxiety and lupus and a past ocular history of glaucoma presented for further evaluation. The patient had no known allergies. The patient’s glaucoma medications were dorzolamide–timolol [twice a day (BID) OU] and latanoprost [one time in the evening (QHS) OU]. The patient’s visual acuity with glasses was 20/20 in the OD and 20/20 in the OS. Their refraction value was −3.50 −1.50 × 068 in the OD and −3.50 −2.00 × 095 in the OS. The IOP for this patient was 19 mmHg in the OD and 18 mmHg in the OS. The gonioscopy showed Shaffer grade III angles, with increased pigmentation inferiorly vs. superiorly in both eyes. The patient’s CDR was 0.8/0.8 in the OD and 0.8/0.8 in the OS, with glaucomatous cupping and thinning of the neuroretinal rim (OU). The OCT–ONH revealed an average RNFL thickness of 62 µm and a CD of 0.81 in the OD, and an average RNFL thickness of 72 µm and CD of 0.76 in the OS. The HVF of the OD revealed a supranasal defect, with a VFI of 92% and an MD of −5.07 dB. The HVF of the OS revealed a central nasal defect, a VFI of 95%, and an MD of −2.80 dB.

After discussing treatment options with the patient, they decided to undergo glaucoma surgery entailing clear lens extraction and IOL placement with OMNI viscocanaloplasty and a HYDRUS stent with an adjacent goniotomy in the OD. After the surgery, the patient was placed on prednisolone acetate (QID), ketorolac (TID), and ofloxacin (QID) in the right eye. The patient continued the use of latanoprost (QHS) and dorzolamide–timolol (BID OS). One month post operation, the patient’s intraocular pressure was 19 mmHg in the OD and 15 mmHg in the OS. At this point, we began to taper the patient off prednisolone acetate and ketorolac. Their visual acuity 6 weeks post operation was 20/20 in the OD and 20/25 in the OS. The patient’s intraocular pressure during this visit was stable, at 11 mmHg in the OD and 14 mmHg in the OS. Four months after undergoing glaucoma surgery, their intraocular pressure was 17 mmHg in the OD and 26 mmHg in the OS. At this point, the patient decided to undergo the same glaucoma surgery in her left eye due to the increase in the IOP. One day post operation, the patient’s distance visual acuity was 20/25 in the OD and 20/25 in the OS. The intraocular pressure was noted to be 13 mmHg in the OD and 14 mmHg in the OS. Three weeks post operation, the patient’s visual acuity was 20/25 in the OD, 20/25 in the OS, and 20/20 in OU. The intraocular pressure was found to be 14 mmHg in the OD and 14 mmHg in the OS. Four months post operation, her IOP was 12 mmHg in the OD and 10 mmHg in the OS and she was taking no medications. The HVF of the OD revealed a VFI of 91% and an MD of −4.93 dB, while the HVF of the OS revealed a VFI of 96% and an MD of −4.07 dB. The visual acuity was 20/20 in the OD and 20/20 in the OS. The refraction value was 0.00 −1.50 × 069 in the OD and +0.25 −1.00 × 093 in the OS. This represented a 3-diopter improvement in spherical equivalence.

## Case study 3

A 49-year-old black female patient with suspected glaucoma, in addition to diabetes and hypercholesterolemia, presented for further evaluation. The visual acuities for distance were 20/20 in the OD and 20/20 in the OS. The refraction for this patient was +2.25 SPH in the OD and +2.25 −0.50 × 084 in the OS. The IOP for this patient was 15 mmHg in the OD and 15 mmHg in the OS. The gonioscopy showed Shaffer grade III angles, with greater pigmentation observed inferiorly than superiorly. The CDR was 0.55/0.55 in the OD and 0.55/0.55 in the OS, with thinning of the inferior neuroretinal rim also observed. The HVF of the OD showed a VFI of 90% and an MD of –4.07, and that of the OS showed a VFI of 87% and an MD of –4.77. The OCT–ONH test revealed an average RNFL thickness of 89 µm in the OD and 79 µm in the OS. The corneal pachymetry showed a central corneal thickness of 497 µm in the OD and 492 µm in the OS. The patient was diagnosed with mild POAG. The patient was placed on latanoprost (QHS OU).

To reduce the need to take eye drops for the rest of their life, the patient provided informed consent and agreed to undergo glaucoma surgery entailing clear lens extraction and IOL placement with OMNI viscocanaloplasty and a HYDRUS stent with an adjacent goniotomy in the left eye. Following surgery, the patient was placed on prednisolone acetate (QID), ketorolac (TID), and ofloxacin (QID) in the left eye, while discontinuing latanoprost in the left eye. One day post operation, the patient’s unaided visual acuity for distance was 20/60 in the OD and 20/20 in the OS. The measured intraocular pressure was 14 mmHg in the OD and 8 mmHg in the OS. The patient continued taking the postoperative medications for 3 weeks and then returned for follow-up. At the 3-week follow-up, the patient’s best corrected visual acuity was 20/20 in both the OD and the OS. The patient’s IOP was 14 mmHg in the OD and 14 mmHg in the OS. The refraction value was −0.50 −0.50 × 053 OS. This represented a 2-diopter improvement in the spherical equivalence. Ofloxacin was discontinued, and prednisolone acetate and ketorolac were tapered at this point. The patient was to continue taking latanoprost (QHS OD). The patient returned for follow-up at 6 months, at which point it was found that their IOP was stable, at 14 mmHg in the OD and 14 mmHg in the OS.

## Case study 4

A 47-year-old black male patient presented with a history of glaucoma. The patient was taking latanoprost (QHS OU), dorzolamide (BID OU), and brimonidine (TID OS). The patient’s visual acuity was 20/20 in the OD and 20/20 in the OS with spectacle correction. The IOP was 17 mmHg in the OD and 17 mmHg in the OS. The refraction value was +0.50 −1.25 × 003 in the OD and +0.25 −1.75 × 003 in the OS. The dark-room gonioscopy showed grade II Shaffer angles that were narrowly occludable. The fundus exam revealed glaucomatous cupping in both eyes and an inferior notch in the OS. The CDR was 0.85/0.85 in the OD and 0.85/0.85 in the OS. The pachymetry test for this patient revealed a corneal thickness of 504 µm in the OD and 499 µm in the OS. The OCT–ONH showed an average RNFL thickness of 80 µm in the OD and 76 µm in the OS. The visual field testing for this patient revealed a VFI of 91% and an MD of −3.58 dB in the OD, and a VFI of 93% with an MD of −4.57 dB in the OS.

The patient provided informed consent and agreed to undergo surgery entailing a clear lens extraction and IOL placement, a HYDRUS stent, and OMNI canaloplasty in the right eye. After the surgery, the patient was placed on prednisolone acetate (QID), ketorolac (TID), and ofloxacin (QID) in the right eye. Five months post operation, the patient’s best corrected visual acuity was 20/20 in the OD and 20/20 in the OS. The patient’s IOP measurement was 14 mmHg in the OD and 14 mmHg in the OS. The visual field test for this patient revealed a VFI of 94% with an MD of −4 dB in the OD and a VFI of 89% with an MD of −8 dB in the OS.

The patient decided to undergo glaucoma surgery in the left eye, which entailed clear lens extraction and IOL placement with OMNI viscocanaloplasty and a HYDRUS stent with adjacent goniotomy. After surgery, the patient was placed on prednisolone acetate (QID), ketorolac (TID), and ofloxacin (QID) in the left eye. On the first day post operation, the patient’s visual acuity was 20/20 in the OD and 20/50 in the OS. The IOP for this patient was 9 mmHg in the OD and 8 mmHg in the OS. Three weeks post operation, the patient’s visual acuity was stable, at 20/20 in the OD and 20/20 in the OS. The final refraction value was –1.00 −1.00 × 173 in the OD and –0.50 −1.50 × 010 in the OS. The patient’s IOP was stable, at 12 mmHg in the OD and 13 mmHg in the OS. At this point, the patient was tapered off prednisolone acetate and ketorolac.

At the 6-month follow-up, the patient’s visual acuity was 20/20 in both eyes. While on latanoprost (QHS OU), their IOP was stable, at 12 mmHg in the OD and 13 mmHg in the OS. This resulted in a decrease of glaucoma medications needed, from two to one in the right eye, and from three to one in the left eye. The HVF revealed a VFI of 96% with an MD of –5.73 dB in the OD, and a VFI of 93% with an MD of –6.88 dB in the OS. The fundus photos taken revealed stable CDRs.

## Discussion

**Figure 3:**
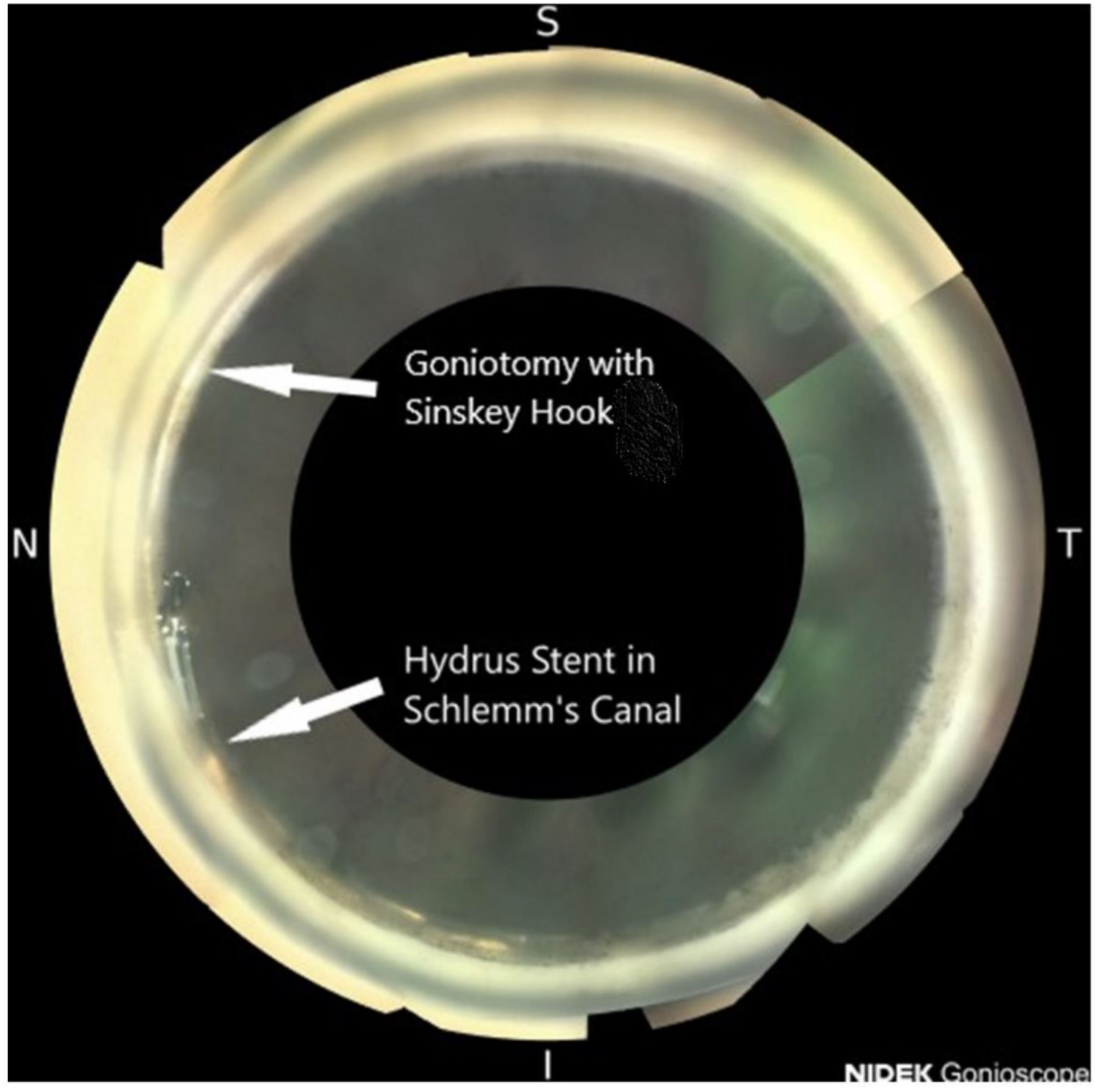
Open angle with a HYDRUS microstent and an adjacent goniotomy: NIDEK GS-1 gonioscopic image.

This is the first case series reporting the combination of a clear lens extraction and IOL placement, OMNI canaloplasty, and a HYDRUS microstent with an adjacent goniotomy (**Figure 3**). All patients achieved lower IOP and stable vision with less refractive error. The patients also experienced improved visual fields, clearer vision, and more open angles. The enlarging lens is one of the most researched hypotheses as a possible cause of glaucoma (10). The removal of the enlarged lens and restoration of aqueous outflow via Schlemm’s canal in younger patients with this combined MIGS is effective in this case series (11).

In this study, we reported four cases of patients with glaucoma with clear lenses whose mean age was 44.5 years. All the patients had excessive pigmentation in the inferior angle compared with the superior angle due to pigment liberation from the enlarged lens rubbing against the posterior iris. All the patients had a mean reduction in IOP from 17 mmHg preoperatively to 12.7 mmHg postoperatively (**Table 1**). The mean number of medications in the patients’ preoperative regimen was 2.2, compared with 0.3 in their postoperative regimen. All patients had stable vision and relatively stable refractions. This surgical treatment also reduce patients’ diurnal intraocular pressure, visual field progression, and potential need for additional glaucoma surgery in the future (25). No complications were noted.

**Table 1:** Pre-operative and post-operative intraocular pressure and medications.

	Pre operation		Post operation	
	IOP(mmHG)	Number of medications	IOP(mmHG)	Number of medications
Average	17.83	2	12.67	0.33
Standard deviation	4.36	1.26	1.75	0.52

A recent study by Okuda showed that patients who underwent cataract extraction and MIGS with a microhook had a higher success rate when using topical glaucoma medications for less than 4.5 years than those who used eye drops for more than 4.5 years (26). The potential reasons for this include the fact that medications containing benzalkonium chloride can be toxic to the trabecular meshwork. A delay in surgery can also compound the pigment obstruction at the trabecular meshwork, the further narrowing of Schlemm’s canal, and the herniation of the collector channels affecting the distal outflow of aqueous humor.

The number of black patients who undergo cataract surgery is 30% less than that of white patients in the United States, and globally, black patients have decreased access to cataract surgery due to a history of slavery, segregation, discrimination, and colonialism (27, 28). Clear lens extraction with IOL placement combined with MIGS in open-angle glaucoma and narrow-angle glaucoma patients resulted in the lowering of IOP. These procedures have an excellent safety profile, employing modern uncomplicated surgical techniques, and can help to reduce the rates of blindness in this population. This lowering of intraocular pressure has been shown to reduce the need for medication and visual field progression over time (20, 29). The target IOP in all of these patients with mild glaucoma was 15 mmHg, and we were able to achieve this. In patients with advanced glaucoma, the target IOP is closer to 8 mmHg–12 mmHg, and achieving this often requires that a trabeculectomy be performed.

No complications were noted in this series. All the surgeries were performed by an experienced cataract and glaucoma surgeon, Daniel Laroche, MD. It is important that as few complications as possible arise as a result of surgery. Potential complications such as hyphema, IOP spikes, and corneal edema, were not seen in this series. This is an initial case series, and the participant number was too small to evaluate a potential synergy between the two procedures. The limitations of this study include the small series and the retrospective potential for bias. Further research with a larger series and prospective trial should be performed. A longer-term follow-up is also needed.

## Data availability statement

The original contributions presented in the study are included in the article/supplementary materials, further inquiries can be directed to the corresponding author/s.

## Ethics statement

The studies involving humans were approved by New York Eye and Ear Infirmary. The studies were conducted in accordance with the local legislation and institutional requirements. Written informed consent for participation was not required from the participants or the participants’ legal guardians/next of kin in accordance with the national legislation and institutional requirements. Written informed consent was obtained from the individual(s) for the publication of any potentially identifiable images or data included in this article.

## Author contributions

DL: Conceptualization, Data curation, Formal analysis, Investigation, Project administration, Visualization, Writing – original draft, Writing – review & editing. AD: Investigation, Visualization, Writing – original draft. CN: Data curation, Formal analysis, Investigation, Visualization, Writing – review & editing.
